# Long-Term Neurological Sequelae Among Severe COVID-19 Patients: A Systematic Review and Meta-Analysis

**DOI:** 10.7759/cureus.29694

**Published:** 2022-09-28

**Authors:** Urvish K Patel, Neev Mehta, Amrapali Patel, Neel Patel, Juan Fernando Ortiz, Mahika Khurana, Eseosa Urhoghide, Akshada Parulekar, Arpita Bhriguvanshi, Nidhi Patel, Anuja Mahesh Mistry, Rutul Patel, Kogulavadanan Arumaithurai, Shamik Shah

**Affiliations:** 1 Public Health and Neurology, Icahn School of Medicine at Mount Sinai, New York, USA; 2 Gastroenterology, Lahey Hospital and Medical Center, Burlington, USA; 3 Epidemiology and Biostatistics, Boston University School of Public Health, Boston, USA; 4 School of Public Health, George Washington University, Washington D.C., USA; 5 Public Health, Icahn School of Medicine at Mount Sinai, New York, USA; 6 Neurology, Universidad San Francisco de Quito, Quito, ECU; 7 Public Health, University of California Berkeley, Berkeley, USA; 8 Internal Medicine, Hudson Regional Hospital, Secaucus, USA; 9 Biology, York University, Toronto, CAN; 10 Child Neurology, Jersey Shore University Medical Center, Neptune City, USA; 11 Medicine, Drexel University College of Medicine, Philadelphia, USA; 12 Neurology, California Institute of Behavioral Neurosciences & Psychology, Fairfield, USA; 13 Internal Medicine, Smt. NHL Municipal Medical College, Ahmedabad, IND; 14 Internal Medicine, Texas Tech University Health Sciences Center El Paso, El Paso, USA; 15 Neurology, Mayo Clinic Health System, Austin, USA; 16 Neurology, Stormont Vail Health, Topeka, USA

**Keywords:** post covid-19 persistent headache and fatigue, covid-19 neuropsychiatric manifestations, post acute sequelae of sars cov-2 infection (pasc), sars-cov-2 and covid-19, covid-19

## Abstract

Few studies have thoroughly evaluated the neuro-invasive effect of severe acute respiratory syndrome coronavirus 2 (SARS-CoV-2) infection, which may contribute to a wide range of sequelae from mild long-term effects like headaches and fatigue to severe events like stroke and arrhythmias. Our study aimed to evaluate the long-term neurological effects of coronavirus disease 2019 (COVID-19) among patients discharged from the hospital.

In this systematic review and meta-analysis, we assessed the long-term neurocognitive effects of COVID-19. Post-COVID-19 neurological sequelae were defined as persistent symptoms of headache, fatigue, myalgia, anosmia, dysgeusia, sleep disturbance, issues with concentration, post-traumatic stress disorder (PTSD), suicidality, and depression long after the acute phase of COVID-19. Data from observational studies describing post-COVID-19 neurocognitive sequelae and severity of COVID-19 from September 1, 2019, to the present were extracted following the Meta-analysis of Observational Studies in Epidemiology (MOOSE) guidelines and Preferred Reporting Items for Systematic Reviews and Meta-Analyses (PRISMA) protocol with a consensus of three independent reviewers. A systematic review was performed for qualitative evaluation and a meta-analysis was performed for quantitative analysis by calculating log odds of COVID-19 neurocognitive sequelae. The odds ratio (OR) and 95% confidence interval (CI) were obtained and forest plots were created using random effects models.

We found seven studies, out of which three were used for quantitative synthesis of evidence. Of the 3,304 post-COVID-19 patients identified, 50.27% were male with a mean age of 56 years; 20.20% had post-COVID-19 symptoms more than two weeks after the acute phase of infection. Among persistence symptoms, neurocognitive symptoms like headache (27.8%), fatigue (26.7%), myalgia (23.14%), anosmia (22.8%), dysgeusia (12.1%), sleep disturbance (63.1%), confusion (32.6%), difficulty to concentrate (22%), and psychiatric symptoms like PTSD (31%), feeling depressed (20%), and suicidality (2%) had a higher prevalence. In meta-analysis, COVID-19 patients with severe symptoms had higher odds of headache (pooled OR: 4.53; 95% CI: 2.37-8.65; p<0.00001; I^2^: 0%) and myalgia (pooled OR: 3.36; 95% CI: 2.71-4.17; p<0.00001; I^2^: 0%). Anosmia, fatigue, and dysgeusia had higher but non-significant odds following COVID-19.

Although we had sufficient data for headache and fatigue to identify higher rates and associations following COVID-19, we could not establish relationships with other post-COVID-19 neurocognitive séqueles. Long-term follow-up may mitigate the neurocognitive effects among COVID-19 patients as these symptoms are also associated with a poor quality of life.

## Introduction and background

Coronavirus disease 2019 (COVID-19), caused by severe acute respiratory syndrome coronavirus 2 (SARS-CoV-2), was first identified in 2019 and has since resulted in a worldwide pandemic. The clinical spectrum of COVID-19 ranges from asymptomatic infection and mild upper respiratory tract illness to severe viral pneumonia with respiratory failure, and even death [1]. Respiratory viral infections have also been associated with neurological symptoms [2]. There is a strong link between neuroinflammation and neurodegenerative disease.

As discussed, neurologic infection and symptoms have been seen as a result of COVID-19 infection. Prolonged immune responses to infection in neural vasculature may promote the onset of diseases like Alzheimer's and other dementias, leading to long-term disability [3]. There have been reports of dementia in older patients after infection with other (non-COVID-19) respiratory viral infections, and hence a potential risk for neurodegeneration exists [3].

Post-COVID-19 patients have displayed a variety of symptoms including headache, stroke, and organ failure [4]. According to a few studies of post-covid symptoms, patients have reported symptoms as late as 20 days after their last negative polymerase chain reaction (PCR) test [4]. The more mild symptoms included muscle and joint pain and fatigue, moderate symptoms included difficulty breathing and migraines, while more severe symptoms included stroke, myocarditis, renal failure, and pulmonary fibrosis. It should be noted that several psychological symptoms have also persisted post-infection and include depression, anxiety, and obsessive-compulsive disorder. The severity of symptoms has been correlated to the length of the infection. The most common symptom reported post-infection is fatigue, as reported by several studies [4].

It is believed that COVID-19’s effects on the nervous system are due to the expression of angiotensin-converting enzyme 2 (ACE2) receptor by the choroid plexus and neocortical neurons that SARS-CoV-2 employs to gain cellular entry, although the expression of ACE2 is low (~2%) [5]. At this point, it is also proposed that their other receptors [basigin, neuropilin 1 (NRP1)] may be expressed in nervous system structures, and serve as points of viral entry. A more plausible route for neurologic entry is via the olfactory system wherein virus particles are endocytosed, and retrogradely delivered to synapses so as to permit infection to the rest of the nervous system [6]. The olfactory route is most plausible as it also explains common neurological deficits (loss of smell and taste) seen in COVID-19 infection. Lastly, already infected immune cells (T cells, neutrophils, monocytes) may transgress the blood-brain barrier, meninges, and choroid plexus, as part of normal function, and bring along virus particles if they had been previously infected in the periphery [5].

The literature on the long-term neurological effects of COVID-19 is limited. In light of this, we conducted a systematic review and a meta-analysis to review the neurological sequelae post-COVID-19 to consolidate the knowledge about the neurological effects of the virus on the nervous system. Our review aimed to gather information from pertinent studies to analyze and compare their results to present a consolidated and analytic assessment of the consequences of the virus on the central and peripheral nervous system.

## Review

Endpoint

The primary aim of this systematic review is to evaluate the long-term neurological effects of symptomatic COVID-19 patients. Post-COVID-19 neurological sequelae were defined as headache, fatigue, myalgia, anosmia, dysgeusia, sleep disturbance, issues with concentration, post-traumatic stress disorder (PTSD), suicidality, and depression. We identified the prevalence of neurological sequelae at the time of the study.

The secondary aim of the study was to evaluate the association between the severity of the symptoms and their association with neurological sequelae. COVID-19 severity ranged from mild symptomatic outpatient to severe symptomatic hospitalized patients and/or ICU admissions and those requiring invasive mechanical ventilation.

Search strategy and selection criteria

We conducted a systematic search on studies published from September 1, 2019, to December 2021 following Meta-analysis of Observational Studies in Epidemiology (MOOSE) guidelines and Preferred Reporting Items for Systematic Reviews and Meta-Analyses (PRISMA) protocol. We used PubMed database for observational studies that described the various long-term neurological sequelae and outcomes of COVID-19 patients by utilizing the following keywords or MeSH terms: "neurological complications"[Title/Abstract] OR "neuroinvasion"[Title/Abstract] OR "headache"[Title/Abstract] OR "fatigue"[Title/Abstract] OR "stroke"[Title/Abstract] OR "myalgia"[Title/Abstract] OR "epilepsy"[Title/Abstract] OR "guillain barre syndrome"[Title/Abstract] OR "encephalitis"[Title/Abstract] OR "PRES"[Title/Abstract] OR "migraine"[Title/Abstract] OR "dementia"[Title/Abstract] AND "persistent covid 19"[Title/Abstract] OR ("long"[All Fields] AND "term covid 19"[Title/Abstract]) OR "post covid syndrome"[Title/Abstract] OR (("Post"[All Fields] AND ("COVID-19"[All Fields] OR "COVID-19"[MeSH Terms] OR "covid 19 vaccines"[All Fields] OR "covid 19 vaccines"[MeSH Terms] OR "covid 19 serotherapy"[All Fields] OR "covid 19 serotherapy"[Supplementary Concept] OR "covid 19 nucleic acid testing"[All Fields] OR "covid 19 nucleic acid testing"[MeSH Terms] OR "covid 19 serological testing"[All Fields] OR "covid 19 serological testing"[MeSH Terms] OR "covid 19 testing"[All Fields] OR "covid 19 testing"[MeSH Terms] OR "sars cov 2"[All Fields] OR "sars cov 2"[MeSH Terms] OR "severe acute respiratory syndrome coronavirus 2"[All Fields] OR "ncov"[All Fields] OR "2019 ncov"[All Fields] OR (("coronavirus"[MeSH Terms] OR "coronavirus"[All Fields] OR "cov"[All Fields]). For the systematic review, non-observational, non-full text, non-English, and animal studies were excluded.

Study selection

**Figure 1:**
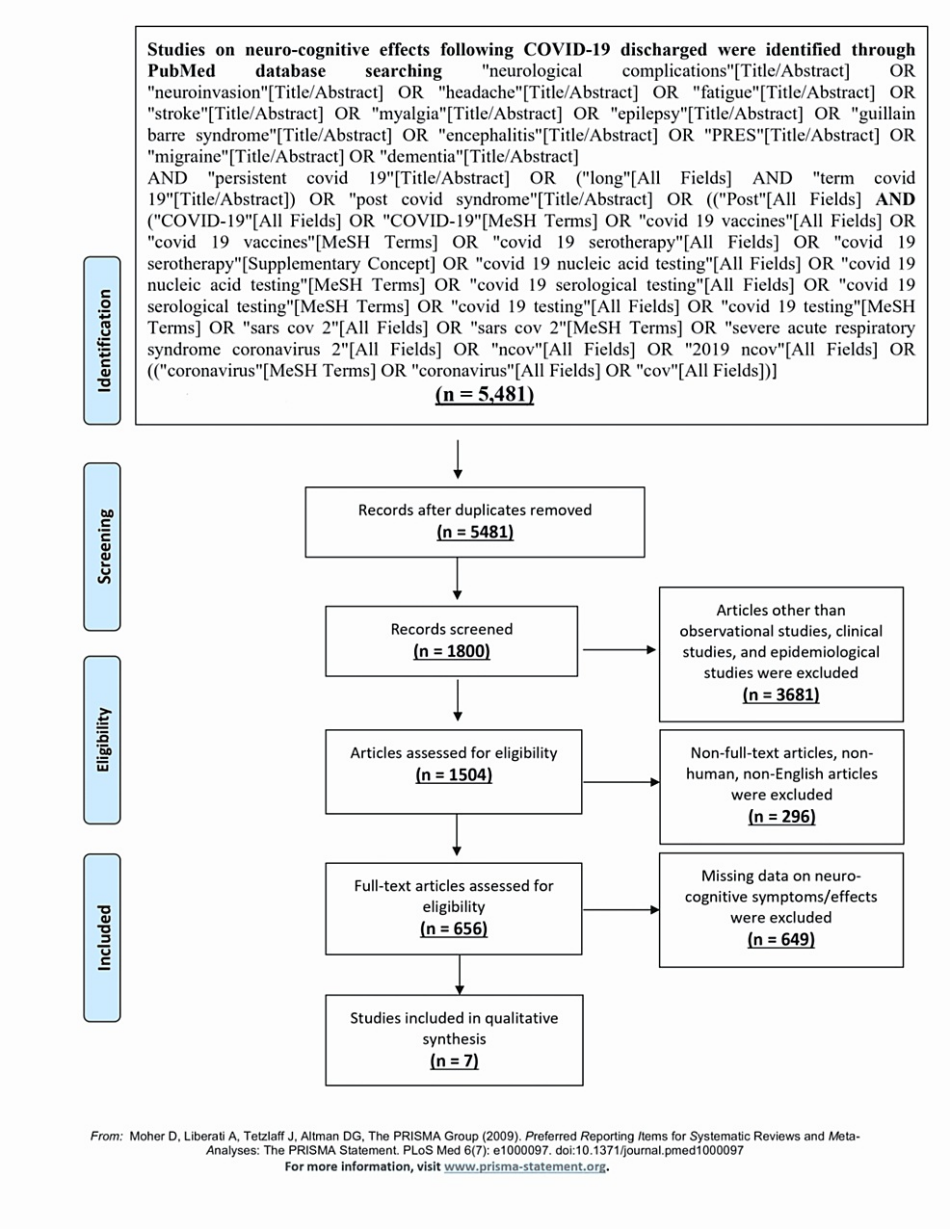
PRISMA flowchart for study selection PRISMA: Preferred Reporting Items for Systematic Reviews and Meta-Analyses

Articles were reviewed for the availability of data on long-term neurologic sequelae in post-COVID-19 patients. Quantitative analysis was conducted on studies that provided details on headache, fatigue, anosmia, dysgeusia, and myalgia. For uniformity of the severity measurement, non-US studies were excluded from the meta-analysis. We independently assessed all identified studies to decide eligibility and resolved any disagreement through consensus. PRISMA flow diagram of the study selection process is described in Figure 1.

Data collection

From the included studies, we extracted the following variables: study details, age, sex, type of COVID-19 severity, follow-up duration, and post-COVID-19 neurological sequelae (headache, fatigue, myalgia, anosmia, dysgeusia, sleep disturbance, concentration, PTSD, suicidality, and depression) using prespecified forms in an Excel sheet by four authors (Amrapali Patel, Juan Ortiz, Neel Patel, and Neev Mehta) by a common consensus. We have presented the study characteristics of individual studies in Table 1.

**Table 1:** Study characteristics showing post-discharge long-term neurological sequelae The severity of COVID-19-associated illness was scored using an ordinal scale developed by a special World Health Organization (WHO) committee for use in randomized multi-center adaptive clinical trials and is based on the site of care (community or hospital) and intensity of oxygen supplementation required SD: standard deviation; OR: odds ratio; CI: confidence interval; RT-PCR: reverse transcriptase polymerase chain reaction test; SONA: online research study management system

Study	Study design	Sample size	Age, years	Male gender (%)	Cases vs. control (column E)	Follow-up duration after discharge, days, mean ±SD/range	Neurocognitive effects/symptoms
Studies considered for meta-analysis (quantitative assessment of point estimate)							
Jacobs et al., USA, Dec 2020 [7]	Prospective cohort study	183	57 (median)	111/183 (60.6%)	Cases: severe COVID-19 disease among hospitalized patients characterized by scores of five (non-invasive ventilation or high-flow oxygen), six (intubation and mechanical ventilation), or seven (ventilation plus additional organ support) vs. controls: mild COVID-19 disease among hospitalized patients characterized by scores of three (no oxygen therapy) or four (oxygen by mask or nasal prongs); subjects were scored using an ordinal scale developed by the WHO committee	35 ± 5 days	Headache - OR: 4.23, 95% CI: 2.15-8.32 (cases: 23/59 vs. control: 24/183); fatigue - OR: 0.93, 95% CI: 0.60-1.44 (cases: 82/149 vs. control 104/183); anosmia - OR: 1.96, 95% CI: 0.99-3.89 (cases: 17/65 vs. control: 28/183); myalgia - OR: 4.0498, 95% CI: 2.28-7.19 (cases:39/77 vs. control: 37/183); dysgeusia - OR: 1.0549, 95% CI :0.56-1.98 (cases: 18/79 vs. control: 40/183)
Fisher et al., USA, Jan 2021 [8]	Matched case-control study	320	40 (median)	151/320 (47.18%)	Cases: positive RT-PCR-confirmed SARS‐CoV‐2 infection (157) vs. control: negative RT-PCR-confirmed SARS‐CoV‐2 infection (163) at outpatient facilities	Interviews were conducted 15-52 days (median, 22 days) after the onset of symptoms	Fatigue - OR: 3.30, 95% CI: 2.60‐4.30 (cases: 121/157 vs. control: 91/163); anosmia - OR: 32.40, 95% CI: 12.60‐83.10 (cases: 98/157 vs. control 9/163); myalgia - OR: 3.30, 95% CI: 2.60‐4.30 (cases: 121/157 vs. control: 91/163)
Walsh-Messinger et al., USA, Nov 2020 [9]	Case-control study	148	19.86 (median)	1/22 (4.54%)	Cases: participants who reported with post-COVID-19 syndrome defined as protracted symptoms ≥28 days (n=22) vs. control A: participants who were never diagnosed with COVID-19 (n=58), and control B: participants who fully recovered (n=21)	Participants were recruited through SONA, an online research study management system; completed the online study about COVID-19 testing, symptoms, course of illness, treatment, and current functioning between October 7 and November 11, 2020, and the self-report measures were administered through Qualtrics (Qualtrics XM, Provo, UT)	Headache - OR: 9.33, 95% CI: 1.03-84.20 (cases: 7/22 vs. control B: 1/21); fatigue - OR: 10.45, 95% CI: 1.9281-56.6384 (cases: 19/22 vs. control B: 10/21); anosmia - OR: 4.09, 95% CI: 1.02-16.27 (cases: 18/22 vs. control B: 11/21); myalgia - OR: 2.33, 95% CI: 0.68-7.94 (cases: 14/22 vs. control: 9/21); dysgeusia - OR: 2.33, 95% CI: 0.68-7.94 (cases: 14/22 vs. control B: 9/21)
Studies considered for systematic review (qualitative assessment)							
Halpin et al., UK, Feb 2021 [10]	Cross-sectional study	100 (68 ward patients and 32 ICU patients)	ICU patients' median age was 58.5 years, and for ward patients, it was 70.5 years	54/100 (54%)	Participants who were RT-PCR-confirmed and treated for COVID-19, out of which 32 participants required treatment in the intensive care unit (ICU group) and 68 participants were managed in hospital wards (ward group), and were screened using a specialist telephone screening tool designed to capture symptoms and report on post-discharge symptoms	Patients treated for COVID-19 were screened by telephone for 29-71 days post-discharge (mean: 48 days)	Fatigue - ICU group: 23/32, ward group: 41/68; OR: 1.6829, 95% CI: 0.68-4.19
Islam et al., Bangladesh, Feb 2021 [11]	Cross-sectional survey	1,002	34.7 (mean)	601/1,002(60%)	A self-reported online pre-tested semi-structured questionnaire was used to collect data over a one-month period involving people who had previously tested positive for COVID-19 and also the individuals who had recovered from COVID-19	Data taken over 1 month (September 11, 2020, to October 13, 2020)	Headache: 80/732 (10.92%), persistent: 8% of 1,002 = 80, ever: 73% = 732; fatigue: 120/810 (14.81%), persistent: 12% of 1,002 = 120, ever: 80.8% = 810; myalgia: 70/531 (13.18%), persistent: 7% of 1,002 = 70, ever: 53% = 531
Huang et al., USA, March 2021 [12]	Retrospective cohort study	1,407	Distributed across all age groups with persons aged 50-59 years (range, ±20 years) representing more than 72% of the long-hauler population. Included those aged ≤18 years in the long haulers group (34 participants, mean age of 9.29 years, 11/34 were aged ≤5 years)	653/1,407 (46%)	A sample of confirmed RT-PCR-positive SARS-CoV-2 patients and those who were never hospitalized for SARS-CoV-2 infection. Patients hospitalized for COVID-19 were excluded	Symptoms assessed at days 0-10 and 61+ among subjects with PCR-confirmed SARS-CoV-2 infection	380/1407 reported persistent symptoms after 60 days; headache: 111/120 (92.54%), 0-10 days: 120, 61+ days: 111; fatigue: 150/122 (123..22%) 0-10 days: 122, 61+ days: 150; anosmia: 25/52 (0.48%), 0-10 days: 52, 61+ days: 25; myalgia: 89/68 (131.59%), 0-10 days: 68, 61+ days: 89; dysgeusia: 47/56 (83.92%), 0-10 days: 56, 61+ days: 47
Carfi et al., Italy, July 2020 [13]	Retrospective cohort study	143	56.5 years (mean)	90/143 (63%)	Participants who were discharged from the hospital after COVID-19 recovery and who tested negative for SARS-CoV-2 were asked to score their quality of life from 0 (worst imaginable health) to 100 (best imaginable health) before COVID-19 and at the time of the visit. A difference of 10 points on the EuroQol visual analog scale was defined as worsened quality of life	Mean of 60 days (SD: 13.6) after symptom onset	Headache: 13/143 (9.1%); fatigue: 76/143 (53.1%); anosmia: 21/143 (14.69%); myalgia: 9/143 (6.29%); dysguesia: 14/143 (9.79%)

Statistical analysis

We used analytical ToolPack in an Excel sheet to calculate the prevalence of post-COVID-19 neuro sequelae. Meta-analysis was performed using Review Manager version 5.4 (The Nordic Cochrane Center, The Cochrane Collaboration). The Mantel-Haenszel formula was used to calculate dichotomous variables to obtain adjusted odds ratios (aORs) along with its 95% confidence interval (CI) to describe the association between COVID-19 severity and neuro-sequelae. Generic-inverse-variance random effects models were used to calculate pooled OR from log odds and events and to obtain forest plots. A p-value <0.05 was considered significant. The bias and quality of the study were evaluated using the Newcastle-Ottawa scale (NOS) (Table 3). The I^2^ statistic (in a meta-analysis, the fraction of variance that is due to heterogeneity is estimated by the statistic I^2^) and funnel plots (graph designed to check for the existence of publication bias) were used to assess statistical heterogeneity.

Results

In our literature search, out of 5,481 searches, 656 full-text articles were assessed for eligibility. After considering eligibility criteria, we included seven observational studies (including three for meta-analysis) with 3,304 confirmed cases of COVID-19. Of note, 50.27% of the patients were male with a mean age of 56 years (range: 34-70); 20.20% had persistent post-COVID-19 symptoms. Symptoms persisted between 30 and 60 days. Among persistent symptoms, neurocognitive symptoms like headache (27.8%), fatigue (26.7%), myalgia (23.14%), anosmia (22.8%), dysgeusia (12.1%), sleep disturbance (63.1%), confusion (32.6%), difficulty to concentrate (22%), and psychiatric symptoms like PTSD (31%), feeling depressed (20%), and suicidality (2%) had a higher prevalence. We have calculated and presented the OR of individual studies in Table 2. For our meta-analysis, we considered 651 confirmed cases out of 3,304 described above, having appropriate details for analysis from Fisher et al., Jacobs et al., and Walsh-Messinger et al. Data used for the meta-analysis is summarized in Table 2.

**Table 2:** Point estimates for post-discharge long-term neurological sequelae among severe (vs. non-severe) COVID-19 patients aOR: adjusted odds ratio; CI: confidence interval

Study	Study design	Sample size (n=651)	Anosmia	Fatigue	Headache	Dysgeusia	Myalgia
Jacobs et al., USA, Dec 2020 [7]	Prospective cohort study	183	aOR: 1.96; 95% CI: 0.99-3.89	aOR: 0.93; 95% CI: 0.60-1.44	aOR: 4.23; 95% CI: 2.15-8.33	aOR: 1.05; 95% CI: 0.56-1.98	aOR: 4.05; 95% CI: 2.28-7.19
Fisher et al., USA, Jan 2021 [8]	Matched case-control study	320	aOR: 32.40; 95% CI: 2.60‐83.32	aOR: 3.30; 95% CI: 2.60‐4.19	Not estimated	aOR: 32.40; 95% CI: 12.60‐83.32	aOR: 3.30; 95% CI: 2.60‐4.19
Walsh-Messinger et al., USA, Nov 2020 [9]	Preliminary report, online study	148	aOR: 4.09; 95% CI: 1.03-16.28	aOR: 10.45; 95% CI: 1.93-56.64	aOR: 9.33; 95% CI: 1.03-84.21	aOR: 2.33; 95% CI: 0.69-7.95	aOR: 2.33; 95% CI: 0.69-7.95

Headache

**Figure 2:**
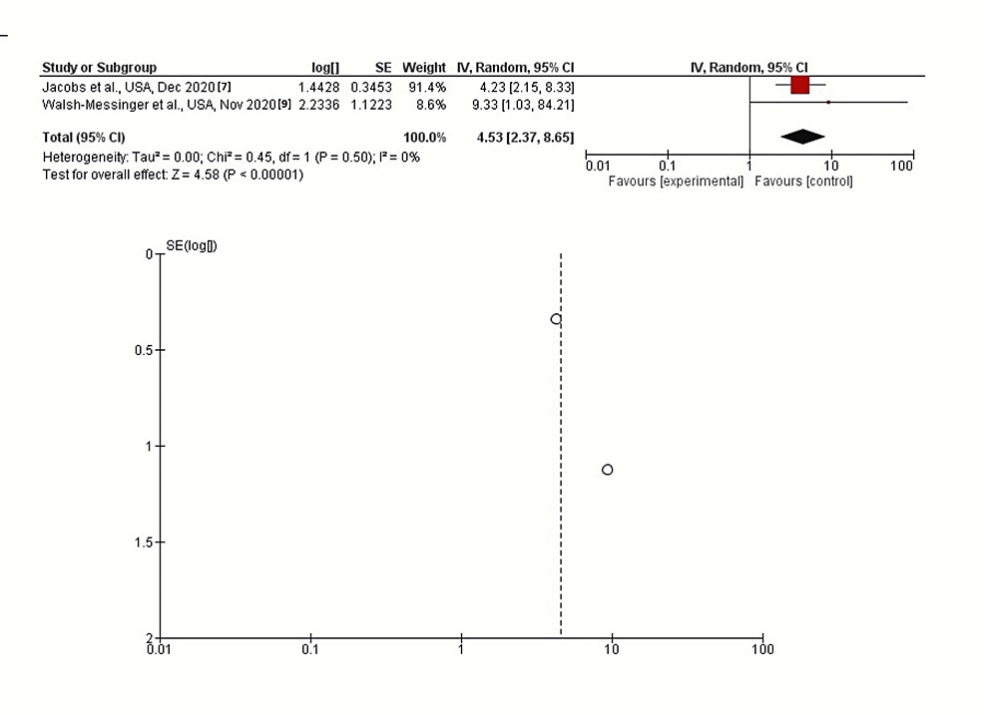
Post-discharge headache as long-term neurological sequelae among severe (vs. non-severe) COVID-19 patients

In inverse-variance, random effects models, severe COVID-19 symptoms were strongly associated with headache with a pooled aOR of 4.53 (95% CI: 2.37-8.65; p<0.00001). Heterogeneity (I^2^) between the study was 0% with a significance of p=0.50 (Figure 2).

Fatigue 

**Figure 3:**
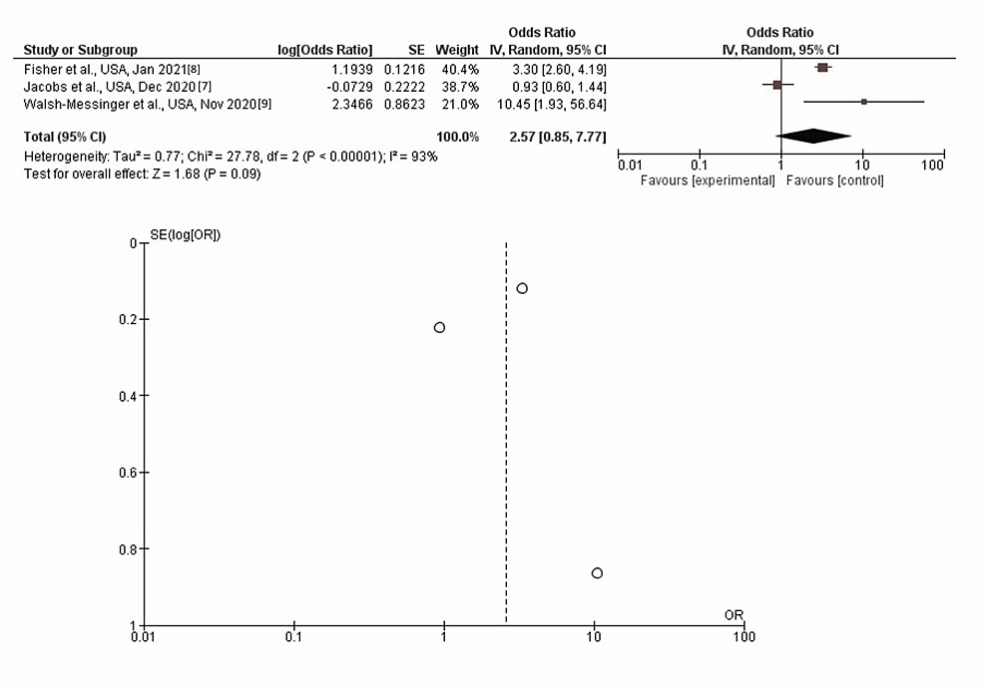
Post-discharge fatigue as long-term neurological sequelae among severe (vs. non-severe) COVID-19 patients

In inverse-variance, random effects models, severe COVID-19 symptoms were not associated with fatigue, with a pooled aOR of 2.57 (95% CI: 0.85-7.77; p=0.09). Heterogeneity (I^2^) between the study was 0% with a significance of p<0.00001 (Figure 3).

Myalgia

**Figure 4:**
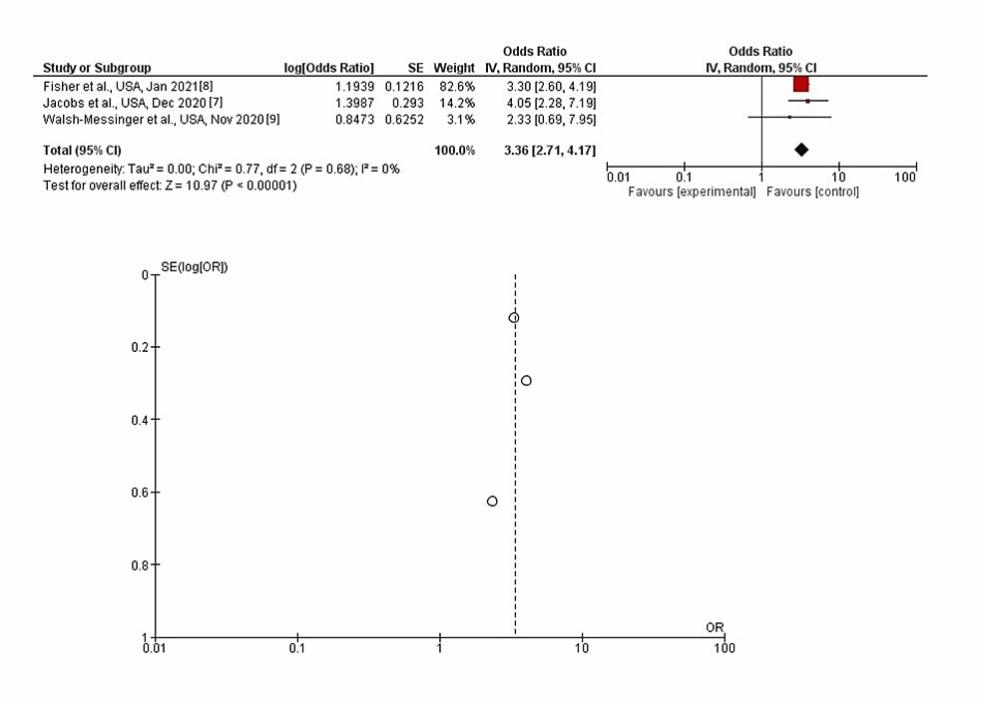
Post-discharge myalgia as long-term neurological sequelae among severe (vs. non-severe) COVID-19 patients

In inverse-variance, random effects models, severe COVID-19 symptoms were strongly associated with myalgia with a pooled aOR of 3.36 (95% CI: 2.71-4.17; p<0.00001). Heterogeneity (I^2^) between the study was 0% with a significance of p=0.68 (Figure 4).

Anosmia

**Figure 5:**
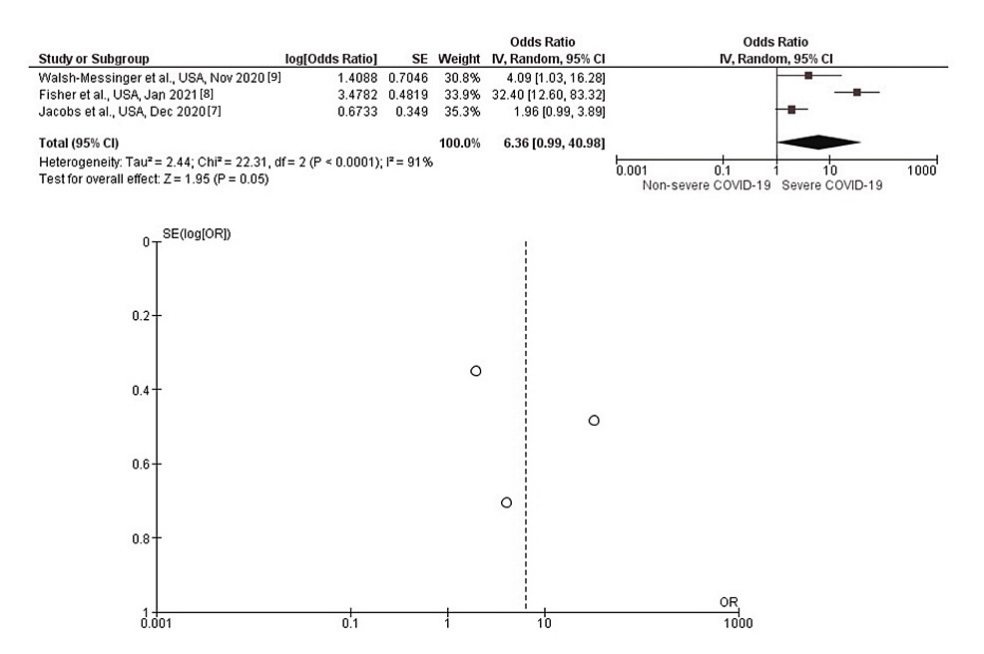
Post-discharge anosmia as long-term neurological sequelae among severe (vs. non-severe) COVID-19 patients

In inverse-variance, random effects models, severe COVID-19 symptoms were strongly associated with anosmia with a pooled aOR of 6.36 (95% CI: 0.99-40.98; p=0.05). Heterogeneity (I^2^) between the study was 0% with a significance of p<0.0001 (Figure 5).

Dysgeusia

**Figure 6:**
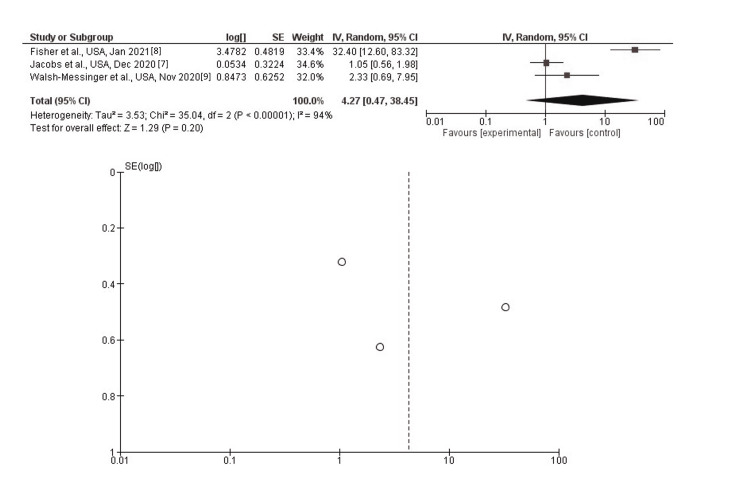
Post-discharge dysgeusia as long-term neurological sequelae among severe (vs. non-severe) COVID-19 patients

In inverse-variance, random effects models, severe COVID-19 symptoms were not associated with dysgeusia, with a pooled aOR of 4.27 (95% CI: 0.47-38.45; p=0.20). Heterogeneity (I^2^) between the study was 0% with a significance of p<0.00001 (Figure 6).

The overall bias of the selected studies is described in Table 3. The majority of the studies had biases ranging from low to moderate, and hence our study has a moderate publication bias.

**Table 3:** Risk of bias of included studies evaluated by Newcastle–Ottawa scale (NOS) For each type of bias mentioned, an asterisk (*) indicates the chance of bias, with subsequently higher chances of bias with each added asterisk (*)

Study	Newcastle–Ottawa Scale			Overall risk of bias
	Selection (max 4*)	Outcome/exposure (max 3*)	Comparability (max 2*)	
Jacobs et al., USA, Dec 2020 [7]	**	**	**	Moderate
Fisher et al., USA, Jan 2021 [8]	***	**	**	Moderate
Walsh-Messinger et al., USA, Nov 2020 [9]	**	**	*	High
Halpin et al., UK, Feb 2021 [10]	***	**	*	Moderate
Islam et al.; Bangladesh, Feb 2021 [11]	***	**	*	Moderate
Huang et al.; USA, Mar 2021 [12]	**	**	*	Moderate
Carfi et al., Italy, Jul 2020 [13]	***	**	*	Moderate

Interpretation

Overall, our study provides insight into various prognostic factors that may correspond with the severity of a COVID-19 infection as well as prognosticate post-COVID-19 neurological sequelae based on the severity of the acute infection. Determining the severity of the infection, through early prognostic neurological markers, may help to guide treatment modalities leading to better patient outcomes. In our study, we found that a severe COVID-19 infection was associated with the following symptoms: headache, myalgia, and anosmia. In addition, we found that fatigue and dysgeusia were symptoms that were not associated with a severe infection. While these findings may initially seem surprising, understanding the pathophysiology behind these symptoms may help to explain why only certain COVID-19 sequelae are associated with severe infection.

Headache

Headaches have been reported in between 11-34% of hospitalized COVID-19 patients. While the mechanism of headaches in COVID-19 patients continues to remain unclear, the most prominent theories hypothesize that either they are the direct activation of the trigeminal nerve by the SARS-CoV-2 virus or activation of the nerve through a secondary system such as pro-inflammatory cytokines [14,15]. While headaches seem to be a relatively common phenomenon among viral infections, we found that headache resulting from SARS-CoV-2 infection was a neurological marker indicative of severe infection [16]. This is perhaps due to the refractory nature of headaches associated with SARS-CoV-2. Unlike most viral-associated headaches, headaches as a result of the SARS-CoV-2 virus have been shown to not improve with the administration of common analgesics and anti-inflammatory medications and tend to have high relapse rates. In addition, it is important to note that patients who develop severe headaches do not usually have a past medical history or any risk factors associated with migraine-type headaches [14]. As a result, the refractory and migraine-like nature of the covid-headache, especially in people who have never experienced a migraine, amplifies the negative feelings of all of the other symptoms associated with the virus - leading patients to experience a more severe form of COVID-19.

Fatigue

Much like headaches, fatigue is another common sequelae of many viral infections. With a SARS-CoV-2 infection, fatigue has been reported in anywhere between 44-70% of all patients [17]. Interestingly, our meta-analysis showed that fatigue has no association with covid severity. Fatigue was once thought to be solely the result of an upregulation of various cytokines and inflammatory molecules, such as interleukin 6 (IL-6), that lead to the disruption of metabolic homeostasis in muscles [18]. However, studies have shown that the degree of pro-inflammatory markers and cytokines do not correspond to the degree of fatigue that patients experience [17]. As a result, COVID-19 fatigue is now thought of as a multifactorial system including central, peripheral, and psychological factors. Because of the lack of correlation between physiological markers (such as inflammatory molecules and cytokines) and fatigue, we suspect that the psychological component, mainly the patient's perceptions, may play a significant role in their experience of fatigue. As a result, fatigue may not only be due to the direct result of the virus, which may help to explain the lack of association between fatigue and infection severity.

Other Neurocognitive Symptoms

Other commonly reported neurological sequelae of COVID-19 include psychological conditions such as depression, anxiety, PTSD, and suicidality. Although the neurophysiology of these cognitive symptoms is not well understood, biopsychosocial elements (biological, psychological, and social aspects) play a key role. A study conducted by Li et al. found that the presence of social support was an independent predictor of a COVID-19 patient's feelings of anxiety, chances of becoming depressed, and their perceived quality of life rating [19]. Moreover, because of the medical precautions preventing families from entering patients' rooms, hospitalized COVID-19 patients have higher chances of developing psychiatric sequelae due to a lack of access to their support systems. Furthermore, the length of hospital stays among COVID-19 patients positively correlated with higher rates of depression and anxiety [20]. Since we primarily focused on the severity of the COVID-19 infection in terms of neurological sequelae, rather than neurological sequelae alone, we did not include these factors in our statistical analysis. However, we highlight them here and in previous paragraphs as examples of other neurological issues that can be attributed to COVID-19.

Quality of Life and COVID-19

Despite not having post-analysis statistical significance, the clinical significance of short and long-term symptoms experienced by patients cannot be overlooked in the quality of life of patients who have recovered from COVID-19 infection [21]. Apart from the neurological sequelae seen above, the pulmonary and extrapulmonary long-term sequelae of other organ systems such as the heart and kidneys add to the apathy of patients who have recovered from COVID-19 [12]. It has been observed that long-term symptoms lead to low physical functioning and abnormal performance of activities of daily living as observed by Belli et al. [22]. Rogers et al. mention that the long-term persistence of symptoms has also been associated with the possibility of the development of depression, PTSD, anxiety, and sleep disorders. In their analysis, measures of quality of life were consistently lower than in the control population and the long-term effects were more pronounced in the social functioning of individuals in comparison to effects on their mental health, thus signifying the broad effects of SARS-CoV-2 virus [23].

Strengths and limitations

The main limitation of our study is the heterogeneity of included projects. Barring the study performed by Jacobs et.al., all studies were retrospective studies. We have also included one cross-sectional survey in our study. The retrospective nature of data collection poses a risk of bias in the final outcome. This is evident from the moderate risk of bias as seen in the Ottawa Scale scores of studies. Secondly, there are variations in definitions of severe vs. non-severe COVID-19 symptoms that might explain the heterogeneity. Thirdly, there was subjective reporting of symptom characteristics in most studies with the possibility of individual variations in the perception of the severity of those symptoms. Due to heterogeneity in studies, many long-term symptoms that were found to be significant in individual studies became non-significant upon calculating the pooled OR. Despite these limitations, our study has performed an analysis on the total sample size of 651 subjects, suggesting the presence of headache and myalgia as long-term sequelae post recovery from COVID-19.

Future directions

Our findings may help in the early triage of high-risk patients and early interventions to prevent long-term morbidity after recovery from the acute phase of COVID-19. Furthermore, active management strategies during the acute phase of COVID-19 to prevent the development of chronic symptoms need to be explored further.

## Conclusions

Our meta-analysis suggests that headache and myalgia are the most common long-term sequelae of COVID-19. These chronic symptoms have added an additional burden on both the patient and the healthcare system, resulting in overall long-term morbidity in patients. Additionally, long-term symptoms lead to poor quality of life and increase the risk of the development of additional somatic and psychiatric disorders. More studies on long-term symptoms of COVID-19 should be designed to better understand the cause of long-term symptoms and to implement early treatment modalities for their prevention.
